# Supplementary material for: Air Quality of Work, Residential, and Traffic Areas during the COVID-19 Lockdown with Insights to Improve Air Quality
Source: Int J Environ Res Public Health. 2022 Jan 10;19(2):727. doi: 10.3390/ijerph19020727 (PMC8775798; doi:10.3390/ijerph19020727)
Supplement: Supplementary file 1 [file ijerph-19-00727-s001.zip › ijerph-1495582-supplementary.pdf]

Supplementary Materials

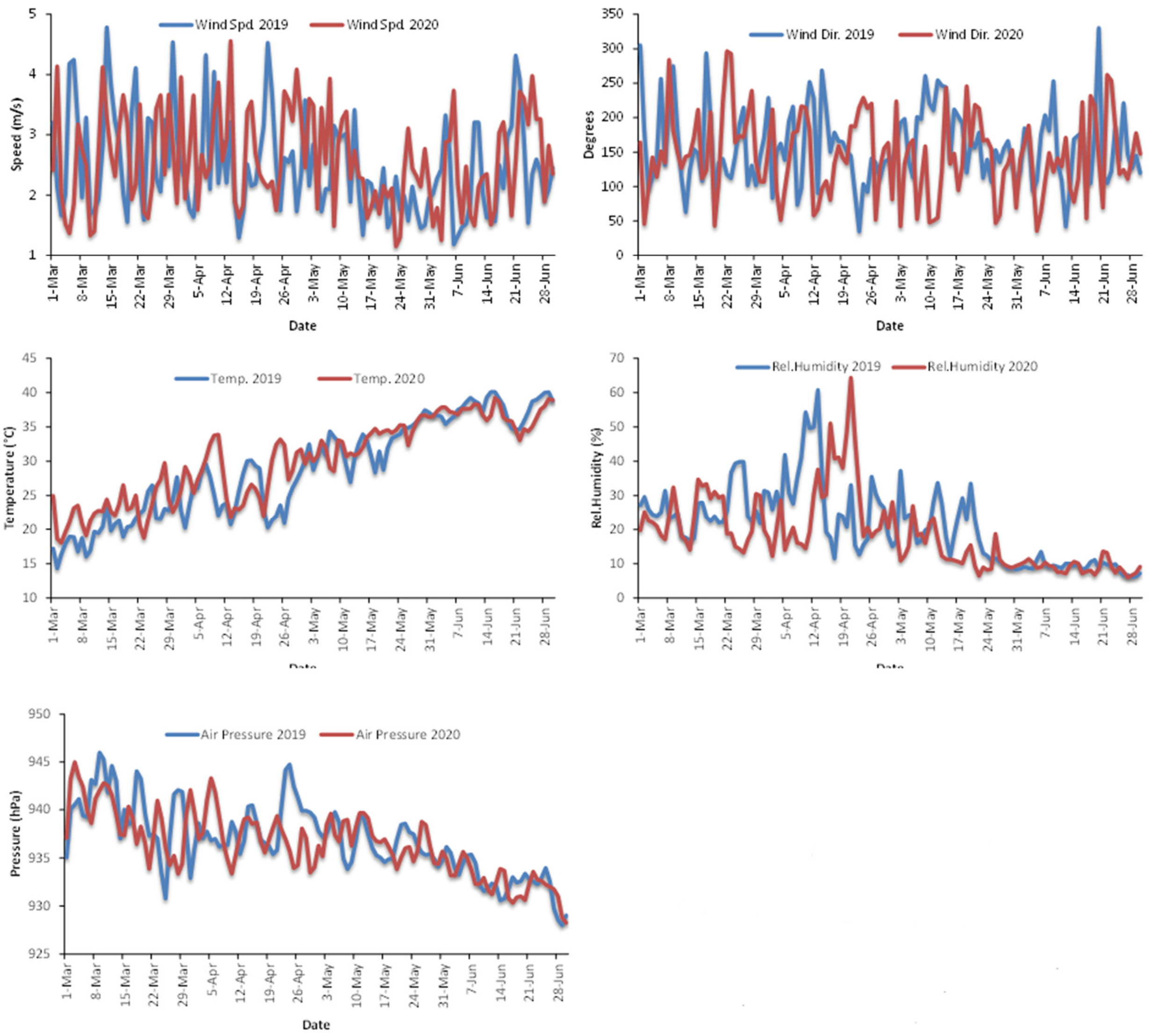

Figure S1. Daily mean temperature, humidity, sea level pressure, wind speed, and wind direction measured from Mar 1 - Jun 30, 2019 and 2020 in Riyadh.

Table S1. Breakpoints for the selected pollutants.

Break points								
**Category**	AQI	O_3 (ppm)		CO (ppm)	SO_2 (ppm)	NO_2 (ppm)	$PM_{2.5}$ (µg/m³)	PM_{10} (µg/m³)
		8-hour	Hourly	Hourly	Hourly	Hourly	Daily	Daily
**Good**	0 – 50	0.000-0.054	-	0.0 – 4.4	0.000-0.035	0.000-0.053	0-12	0-54
**Moderate**	51 – 100	0.055-0.070	-	4.5-9.4	0.036-0.075	0.054-0.100	12.1-35.4	55 – 154
**Unhealthy for sensitive groups**	101 – 150	0.072-0.085	0.125-0.164	9.5-12.4	0.076-0.185	0.101-0.360	35.5-55.4	155-254
**Unhealthy**	151 –200	0.086-0.105	0.165-0.204	12.5-15.4	0.186-0.304	0.361-0.649	55.5-150.4	255- 424
**Very Unhealthy**	201 – 300	0.106-0.200	0.205-0.404	15.5-30.4	-	0.650-1.244	150.5-250.4	355- 424
**Hazardous**	301 – 400	-	0.405-0.504	30.5-40.4	-	1.245-1.644	250.5-350.4	425- 504
**Hazardous**	401 – 500	-	0.505-0.604	40.5-50.4	-	1.645-2.044	350.5-500.4	505-604

Table S2. Daily mean temperature, humidity, sea level pressure, wind speed, and wind direction measured from Mar 1 - Jun 30, 2019 and 2020 in Riyadh.

		Air Pressure (hPa)	Rel. Humidity (%)	Temperature (°C)	Wind speed (m/s)
2019	Average	936.83	21.03	28.94	2.48
	Max	946.01	60.78	40.10	4.78
	Min	928.00	6.20	14.32	1.18
	SD±	3.75	11.17	7.16	0.81
2020	Average	936.43	18.41	30.13	2.58
	Max	945.02	64.35	39.25	4.56
	Min	928.25	6.01	18.04	1.15
	SD±	3.41	10.68	5.92	0.79

	WDIR	Mean wind speed (m/s)				Percent of total (%)
		0-1.37	1.38-3.06	3.06-5.28	>5.28	
2019	N	16	79	135	35	9.10
	NNE	10	147	110	13	9.62
	NE	16	86	34	0	4.67
	ENE	34	99	41	4	6.11
	E	57	71	4	0	4.53
	ESE	61	52	1	0	3.92
	SE	94	232	185	27	18.48
	SSE	82	91	56	8	8.14
	S	55	101	32	4	6.60
	SSW	36	36	1	1	2.54
	SW	24	24	12	1	2.10
	WSW	43	50	32	15	4.81
	W	23	66	52	12	5.26
	WNW	55	68	22	1	5.02
	NW	55	58	11	0	4.26
	NNW	36	94	10	1	4.84
2020	N	8	104	166	19	10.21
	NNE	11	156	203	14	13.20
	NE	14	96	63	1	5.98
	ENE	23	123	92	13	8.63
	E	51	53	4	0	3.71
	ESE	59	48	1	0	3.71
	SE	87	198	157	22	15.95
	SSE	64	98	25	6	6.63
	S	57	95	47	8	7.12
	SSW	25	26	1	0	1.79
	SW	17	18	14	3	1.79
	WSW	18	40	45	16	4.09
	W	33	38	43	1	3.95
	WNW	60	81	6	0	5.05
	NW	51	48	2	0	3.47
	NNW	29	104	4	0	4.71

Table S3. Degree of similarity or discrepancy in the concentrations of air pollutants among the selected residential, traffic, and work sites during the selected study periods.

Period	Pollutant		Residential site (M-station) Concentration	Traffic site (F-station) Concentration	Work site (K-station) Concentration	Residential and work CD	Residential and traffic CD	Traffic and work CD
Lockdown period	CO	Mean	0.20	0.33	0.18	0.50	0.20	0.61
		Max	1.55	3.25	0.81			
		Min	0.01	0.01	0.09			
	NO	Mean	1.66	13.87	5.08	0.36	0.46	0.49
		Max	51.20	275.40	63.90			
		Min	0.10	0.10	0.05			
	NO_2	Mean	13.19	18.68	11.20	0.53	0.33	0.46
		Max	62.10	77.20	92.75			
		Min	1.80	0.70	0.10			
	NO_x	Mean	14.55	30.69	23.58	0.53	0.39	0.48
		Max	110.40	331.10	119.00			
		Min	1.70	0.50	0.10			
	SO_2	Mean	2.11		2.53	0.57	-	-
		Max	16.43	-	30.50			
		Min	0.01		0.30			
	PM_{10}	Mean	179.49	173.80	155.73	0.32	0.26	0.33
		Max	1185.59	1099.35	1292.80			
		Min	44.54	47.95	13.33			
	$PM_{2.5}$	Mean	52.42	49.07	60.98	0.04	0.27	0.09
		Max	306.50	267.03	309.05			
		Min	12.40	15.01	12.48			
Unrestricted periods	CO	Mean	0.33	0.67	0.26	0.40	0.21	0.56
		Max	1.21	3.12	0.89			
		Min	0.02	0.02	0.10			
	NO	Mean	4.75	32.01	7.08	0.12	0.43	0.49
		Max	81.50	310.90	90.15			
		Min	0.10	0.10	0.10			
	NO_2	Mean	24.48	29.92	20.31	0.56	0.17	0.57
		Max	69.90	73.50	60.65			
		Min	6.70	7.00	0.15			
	NO_x	Mean	29.10	61.67	27.30	0.56	0.23	0.66
		Max	143.50	358.10	133.45			
		Min	6.90	8.20	0.10			
	SO_2	Mean	1.54	-	2.70	0.60	-	-
		Max	9.58	-	33.85			
		Min	0.06	-	0.65			
	PM_{10}	Mean	235.29	206.97	144.80	0.27	0.32	0.22
		Max	960.08	542.20	414.35			
		Min	65.55	40.01	74.50			
	$PM_{2.5}$	Mean	60.09	57.71	33.62	0.24	0.34	0.33
		Max	166.48	159.97	90.90			
		Min	17.75	6.11	15.22			

Table S4. GAMEP and USEPA air pollutant standards.

Pollutant	GAMEP Std.	USEPA Std.	Averaging Time
**Nitrogen Dioxide (NO_2)**	0.35 ppm	0.1 ppm	1 h
	0.05 ppm	0.053 ppm	Annual
**Ozone (O_3)**	0.12 ppm	0.12 ppm	1 h
	0.08 ppm	0.075 ppm	8 h
**Particulate Matter (PM_{10})**	340 µg/m^3	150 µg/m^3	24 h
	80 µg/m^3	---	Annual
**Particulate Matter ($PM_{2.5}$)**	35 µg/m^3	35 µg/m^3	2 h
	15 µg/m^3	15 µg/m^3	Annual
**Sulfur Dioxide (SO_2)**	0.28 ppm	0.075 ppm	1 h
	0.14 ppm	0.14 ppm	24 h
	0.03 ppm	0.03 ppm	Annual
**Carbon Monoxide (CO)**	32 ppm	35 ppm	1 h
	8.1 ppm	9 ppm	8 h
